# Comparison of cardiovascular magnetic resonance with real-time three-dimensional echocardiography and the right ventricular automated systolic index in the assessment of the right ventricular function

**DOI:** 10.1186/1532-429X-15-S1-E73

**Published:** 2013-01-30

**Authors:** Florian Andre, Sebastian Greiner, Cihan Celik, Mohamed A Abdelrazek, Maria Fernanda Braggion Santos, Dirk Lossnitzer

**Affiliations:** 1 Department of Cardiology, University of Heidelberg, Heidelberg, Germany; 2 Radiology, Cairo university, Faculty of Medicine, Cairo, Egypt; 3 School of Medicine of Ribeirao Preto University of Sao Paulo, Sao Paulo, Brazil

## Background

The right ventricular (RV) function has an important diagnostic value in many cardiopulmonary diseases and is a predictor for the long-term outcome. Cardiovascular magnetic resonance (CMR) is the gold-standard for the RV quantification as the complex anatomy of the RV, i.e. its crescentic shape, impedes reliable measurement by two-dimensional echocardiography (EC). Yet, CMR or real-time 3D echocardiography (RT3DE) measurements are time consuming. Recently, a novel EC parameter, the RV automated systolic index (RV-ASI), has been introduced which employs semi-automated whole-cycle endocardial border detection and calculates volume changes based on the sum-of-discs method (modified Simpson's rule). In this study we evaluate the measurement agreements of two novel EC parameters, a) the RV-ASI and b) the RT3DE, with the reference standard CMR.

## Methods

We studied 25 patients with different cardiopulmonary diseases (coronary artery disease with preserved left ventricular function (n=4), ischemic cardiomyopathy (n=4), dilated cardiomyopathy (n=10), hypertrophic cardiomyopathy (n=1), cardiac amyloidosis (n=2), pulmonary hypertension (n=4)) and 15 healthy subjects. CMR imaging was performed on a 1.5 T whole-body MRI-scanner applying a cine SSFP sequence with parallel imaging. EC was performed within 30 min with a commercially available ultrasound machine (GE Vivid E9) including RT3DE and the measurement of the RV-ASI. Student's t-test or Mann-Whitney-Wilcoxon-test respectively as well as a regression analysis and a Bland-Altman-plot were performed. Receiver operator characteristics were calculated for RV-ASI compared to CMR RV ejection fraction (RV-EF). A p<0.05 was regarded as statistically significant.

## Results

The RV measurements could be assessed in 100% of subjects by CMR. RV-ASI was evaluable in 38 of 40 subjects (95 %) by EC. Mean RV-EF measured by CMR was 48±9% compared to 51±10% by RT3DE and a RV-ASI of 52±11%. The correlation between CMR RV-EF and RV-ASI (r=0.74, p<0.0001) as well as between CMR RV-EF and RT3DE RV-EF (r=0.55, p=0.0003) were highly significant. The limits of agreement were ±15.1% for RV-ASI and ±17.4% for RT3DE RV-EF compared to CMR RV-EF. An RV-ASI cut-off value of 52% could differentiate between normal and impaired RV function (AUC=0.92, sensitivity=87%, specificity=93%) in this mixed study population.

## Conclusions

In this study the time-saving RV-ASI method showed good agreement with CMR regarding the quantification of the RV function. Although it does not provide the measurement of absolute systolic and diastolic RV volumes, the RV function can be assessed reliably. As it can be obtained easily, it may be utilised for non-invasive follow-up examinations of patients with cardiopulmonary diseases.

## Funding

none

**Table 1:** Comparison of RT3DE and RV-ASI to CMR

	r	Bias	Limits of agreement	p
RT3DE RV-EF (%)	0.55	+3.1%	±17.4%	0.0003

RV-ASI (%)	0.74	+4.4%	±15.1%	<0.0001

**Figure 1:**
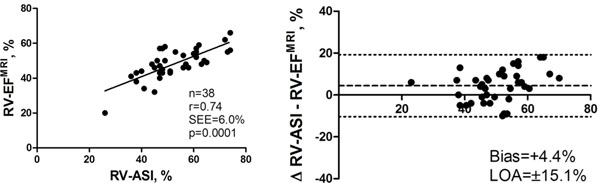
Left: linear regression analysis of RV-ASI and CMR RV-EF. Right: corresponding Bland-Altman-plot of RV-ASI and CMR RV-EF.

